# The Treatment of Chronic Catarrhal Gastritis1Read at the Thirty-eighth Annual Meeting of the Medical Association of Central New York, held at Buffalo, October, 24, 1905.

**Published:** 1906-05

**Authors:** Allen A. Jones

**Affiliations:** Adjunct Professor of Medicine, University of Buffalo


### BUFFALO MEDICAL JOURNAL.

VOL. LXI. MAY, 1906. No. 10

### ORIGINAL COMMUNICATIONS.

INDEX THROUGH

### The Treatment of Chronic Catarrhal Gastritis.[1]

BY ALLEN A. JONES, M. D.

Adjunct Professor of Medicine, University of Buffalo.

WHEN it is possible to improve the state of the circulation in the stomach in those conditions in which it is impaired, there is a possibility of preventing, retarding, or alleviating the consequent catarrhal condition dependent upon congestion. Theoretically, the causal treatment of chronic gastritis is ideal, but, practically, it is difficult of accomplishment. In the instances of pulmonary emphysema and cirrhosis of the liver, it is often impossible to stay the respective pathologic processes. Happily, however, some cases early treated are amenable to considerable amelioration. As illustrations, syphilitic hepatic disease and asthmatic emphysema may be cited. Probably in the case of cardiac disease resulting in passive congestion of the stomach, the best opportunity for casual treatment is offered, as much may be accomplished in the way of circulatory restoration or improvement in all but the most serious and intractable cases.

The etiologic influence of tuberculosis, splenic anemia, nephritis, rheumatism, gout, diabetes and other diseases should be borne in mind, and treatment instituted accordingly. Chronic catarrhal gastritis accompanying other disease of the stomach,—such as atony, ptosis or pyloric stenosis,—is an important phase of the affection, and should be allayed, in as far as possible.

Let me emphasize a phase of gastritis that is not dwelt upon sufficiently, and that is, the importance of infection of the gastric mucosa because of its lowered resistance from functional distress. The stomach is part of the patient, and is acutely sensitive to all influences affecting the whole body or a part of the body. When the brain is fatigued the stomach is also fatigued. When the sympathetic nervous system is exhausted by excessive demand upon it, or by excessive reflex nag and drain, the stomach

1. Read at the Thirty-eighth Annual Meeting of the Medical Association of Central New York, held at Buffalo, October, 24, 1905.

is exhausted and functionally unfit. Trophic depression renders the gastric mucosa vulnerable, and infective organisms are not slow to attack it.

No doubt many instances of gastritis are initiated in early life by ignorant, careless overeating. Most children will eat anything and everything they can get. Many of them, if unrestrained, will eat most of the time. They masticate imperfectly, and overload their stomachs daily with enormous quantities of coarse, indigestible and irritating food. In later life atrocious dietary indiscretions and the abuse of alcohol are frequently to blame for chronic gastric catarrh. Therefore, the first measure of importance in the treatment of the affection is the regulation of the diet. This will be determined by the state of gastric secretion and motion. If secretion is sufficient to develop free hydrochloric acid, more proteids may be allowed than is the case if the free acid is absent. If the total acidity is nearly or quite up to the lower normal limit and is due in proper proportion to combined chloride, albumens may be allowed in considerable quantities. Theoretically, as the secretion is lowered, so should the proteids be restricted, but, practically, it is not best to deprive the patient of proteids too far. Intestinal digestion in most cases is capable of compensating for the gastric failure, and sufficient proteid should be given to meet the requirements of nutrition.

One of the most important points in reference to the diet is as to the mechanical state of the food that is allowed. It should be finely divided and thoroughly masticated. No coarse nor indigestible foods should be allowed. Cereals must be thoroughly cooked, and preferably in a form requiring mastication,—like puffed rice and shredded wheat. In suitable cases malt or takadiastase may be mixed with cereals that are taken in the form of mush,—such as cream of wheat, wheat flake, and wheatlet. Zweibach is preferable to other forms of breadstuff; brown breads are not to be eaten daily, but merely as a change. Vegetables should be strained through a collander or taken in the form of purèes. Raw fruit is not permissible until after considerable improvement has taken place, as it is bulky without containing much nourishment, and it is desirable to supply the needed number of calories in small or moderate bulk. Eggs, always soft, are to be allowed in liberal quantities. Meats should be scraped or finely chopped. Sweetbreads are delicate and nutritious if all fiber is eliminated, and they are nicely cooked with stock and cream with mushroom flavoring. Fried foods should never be given, but fats in the form of butter and cream are necessary to maintain heat and weight.

Milk should be given peptonized until distinct lessening of the catarrh is observed. Condiments should be interdicted ordinarily; but, if secretion is low and but little mucus present, small amounts may be allowed. Tea, coffee, cocoa and chocolate may be given in small quantities, not regularly, but by way of variety. Stewed fruits without the skins and coarser parts, form an excellent relish, and fruit juices also should be allowed quite freely. Care should be taken to avoid particularly articles that are liable to induce food-poisoning, and I interdict all shellfish, cheese, caviar and canned goods for that reason. To conform to the principle of rest for the inflamed organ, it is best that but three meals be given each day, provided at each sufficient food to meet the requirements of nutrition is tolerated; if not, small meals at frequent intervals, according to the German plan, may be ordered.

Next in importance to diet is local treatment of the stomach. Before breakfast or five hours after a meal, lavage should be done. The frequency with which it should be practised should be determined by the amount of mucus present in the contents, by the adequacy of response to diet and treatment, and by the manner in which the patient bears the treatment. There can be no fixed rule to apply to all cases. Too frequent lavage is apt to do more harm than good. It may be well to give several daily treatments and thereafter two or three a week, soon lessening the frequency. Plain water may be used until the stomach is cleansed of food, and then always a saline solution or boric-acid solution should be employed. Weak solutions of sodium bicarb., borax, sodium chloride, or Carlsbad salts are all commendable. At the termination of lavage, distilled water should be employed to clear the organ, and a weak solution of silver nitrate—2 to 4 grains to the ounce introduced through the tube, and allowed to flow out again. There seems no better application than silver, but hydrastis should be employed at some sittings, and resorcin solution 5 grs. ℥viii— at others. Although unpleasant in odor, there is no agent that excels ichthyol water as a lavement. With resorcin it seems to exert a regenerating effect upon epithelia, and is an excellent antiseptic. If fermentation is found in the stomach contents, a few grains of salicylic acid solution may be employed as a wash, and we use a weak solution—2 grs. to the pint—of potassium permangate quite frequently.

If, in addition to these measures, the patient has plenty of rest and recreation out of doors, improvement may be effected without resort to drugs. If there is constipation, high enemata of ichthyol and boroglyceride should be employed daily for a time; less frequently thereafter. Caution should be exercised in the use of cathartics per orem, as they are nearly all irritating. Carlsbad water is of use in cases in which gastric motility is active. Small doses of aloes or cascara suffice for atonic cases, though abdominal Faradization and massage should be thoroughly tried.

It is a mistake to place too much dependence upon drugs in the treatment of this affection. Those that act as stomachics,—such as nux vomica, gentian, cinchona, quassia, calumba and orexin,—are useful to stimulate the appetite and excite gastric secretion. Tissue remedies—such as iron and the glycerophosphates—are of value. During the course of the disease many periods of exacerbation or what might be termed waves of increased or fresh gastritis, supervene and in the treatment of these bismuth subcarbonate, sodium bicarbonate and calcined magnesia are very advantageous. An inflamed and irritable gastric mucosa is intolerant of acids, and alkaline sedative remedies, exhibited in liberal quantities midway between meals and at bedtime, are grateful and beneficial.

If the patient be free from overwork and care; if he lives out of doors, following gentle woodland pursuits; if he embraces the benefits to be obtained at the seashore and in the mountains; if he deserts badly lighted and badly ventilated rooms and offices for horseback and golf; if he balances these with adequate rest and sleep, he will enhance his chances of recovery.

436 Franklin Street.

